# Accurate Classification of Biological and non-Biological Interfaces in Protein Crystal Structures using Subtle Covariation Signals

**DOI:** 10.1038/s41598-019-48913-8

**Published:** 2019-08-30

**Authors:** Yoshinori Fukasawa, Kentaro Tomii

**Affiliations:** 1 Artificial Intelligence Research Center, National Institute of Advanced Industrial Science and Technology (AIST), 2-4-7 Aomi, Koto-ku, Tokyo, 135-0064 Japan; 2 Biotechnology Research Institute for Drug Discovery, National Institute of Advanced Industrial Science and Technology (AIST), 2-4-7 Aomi, Koto-ku, Tokyo, 135-0064 Japan; 3 AIST-Tokyo Tech Real World Big-Data Computation Open Innovation Laboratory, Tokyo, 152-8550 Japan

**Keywords:** Biophysics, Computational biology and bioinformatics, Machine learning

## Abstract

Proteins often work as oligomers or multimers *in vivo*. Therefore, elucidating their oligomeric or multimeric form (quaternary structure) is crucially important to ascertain their function. X-ray crystal structures of numerous proteins have been accumulated, providing information related to their biological units. Extracting information of biological units from protein crystal structures represents a meaningful task for modern biology. Nevertheless, although many methods have been proposed for identifying biological units appearing in protein crystal structures, it is difficult to distinguish biological protein–protein interfaces from crystallographic ones. Therefore, our simple but highly accurate classifier was developed to infer biological units in protein crystal structures using large amounts of protein sequence information and a modern contact prediction method to exploit covariation signals (CSs) in proteins. We demonstrate that our proposed method is promising even for weak signals of biological interfaces. We also discuss the relation between classification accuracy and conservation of biological units, and illustrate how the selection of sequences included in multiple sequence alignments as sources for obtaining CSs affects the results. With increased amounts of sequence data, the proposed method is expected to become increasingly useful.

## Introduction

Protein crystal structures are valuable resources for elucidating functional information of proteins because they contain information related to their functional forms, i.e., biological assemblies (biological units). One important characteristic of protein crystal structures is that the biological units can exist in a crystal lattice that also includes non-biological interfaces. It remains challenging to discern biological contacts from crystal contacts because of the broad overlap of their mutual interface properties, especially biological interfaces and large crystallographic interfaces^1^.

In recent decades, various methods and analyses have been reported for identifying biological units in protein crystal structures. Carugo and Argos statistically analyzed differences between biological and crystal contacts^2^. A pioneering formulation of such features was used in the Protein Quaternary Structure (PQS)^3^ file server. The Protein Interfaces and Assemblies (PITA) score characterizes interfaces according to their contact size and chemical complementarity. PITA also builds up an assembly using a graphic description of the protein quaternary structure and scored interfaces^4^. The atom-based potential of mean force (PMFScore), which incorporates factors such as packing density, contact size, and geometric complementarity^5^ of interfaces, was proposed for distinguishing biological and crystal contacts. That same year, PreBI, based on the sum of the three complementarities (electrostatic potential, hydrophobicity, and interface shape) and contact size was also introduced^6^. Later, COMP was used to identify crystal contacts using contact size and a linear combination of the same three complementarities^7^. Currently, the *de facto* standard method in this field is PISA, which calculates the interface stability and entropy of dissociation^8^. Investigations of a beneficial single parameter for interface classification have also been conducted intensively. Reportedly, local atomic density and residue propensity are useful classification parameters^9^. Additionally, mobility in interfaces is a good feature to discriminate biological contacts from crystal contacts. Liu and colleagues suggested novel features based on B-factor^10^. Furthermore, for this task, DynaFace used normal mode analysis with a Gaussian network model^11^. In addition to physicochemical features, evolutionary information has also been used heavily for this task. Commonly observed interfaces in different crystal forms, especially commonly observed contacts among homologous structures, tend to be biological contacts^12,13^ because biological contacts tend to be conserved evolutionarily among homologs. A pioneering study revealed that interface residues tend to be conserved rather than exposed surface residues^14^. Different evolutionary biases of interface and non-interface residues have also been reported for homodimeric interfaces^15^. Within the biological interfaces, the degree of conservation varies by residue. A few buried residues, comprising the so-called “core”, are more conserved than surrounding interface residues^16^. Work in this area was improved further with a new classifier, EPPIC, which uses the Shannon entropy ratio based only on fairly similar homologous sequences. Actually, EPPIC performed well with the new difficult dataset^17^.

Although some parameters such as contact size and geometric complementarity work to some degree, each parameter alone is not always sufficient for interface classification. Therefore, a combination of the interface parameters has been used to characterize the biological interface. Among such combinatory approaches, NOXclass is a pioneering work. It uses feature sets of three types for support vector machine (SVM) models^18^. Actually, DiMoVo depends on an SVM model with different feature sets. The present study particularly addresses features generated by Voronoi tessellation^19^. Luo *et al*. and Da Silva *et al*. respectively constructed random forest (RF) models based on unique features^20,21^. Elez *et al*. applied and assessed multiple machine learning methods^22^. Its web-server version, PRODIGY-CRYSTAL, was recently released^23^.

The amount of sequence data has increased rapidly during the last decade. Those large amounts of data have enabled contact prediction using covariation signals (CSs) calculated from multiple sequence alignments (MSAs) at a more precise level. Formulations and applications of contact prediction are currently an intensively researched topic in the field of protein science. Particularly, recent progress of contact prediction has often been reported in the area of contact prediction of monomeric proteins’ intrachain contacts. For instance, methods based on estimation of an inverse covariance matrix were breakthroughs that estimated direct coupling sites^24,25^, whereas a pseudo-likelihood approach demonstrated higher accuracy^26,27^. Furthermore, recent advancements of sequencing technology and the accumulation of prokaryotic sequences have enabled the prediction of protein structures on a larger scale^28^. Research of contact prediction using machine learning has also been surveyed intensely^29^. Although contact prediction is successful for intrachain contacts, contact prediction has also been applied to build up a complex via determination of interchain contacts^30–32^. Recently, contact prediction has been applied to predict homodimer forms^33^. The applicability of contact prediction for protein interactions appears promising, but only a few complexes were tested, perhaps because of weak signals in numerous complexes. We applied contact prediction for the interface classification problem in protein crystals where actual contacts are already given, which demonstrated that using features based on CSs is promising for this field of study. This study also elucidates differences between the contact prediction of intrachain and that of the interaction interface.

## Results

### Viability of using CSs with current sequence data

**Figure 1:**
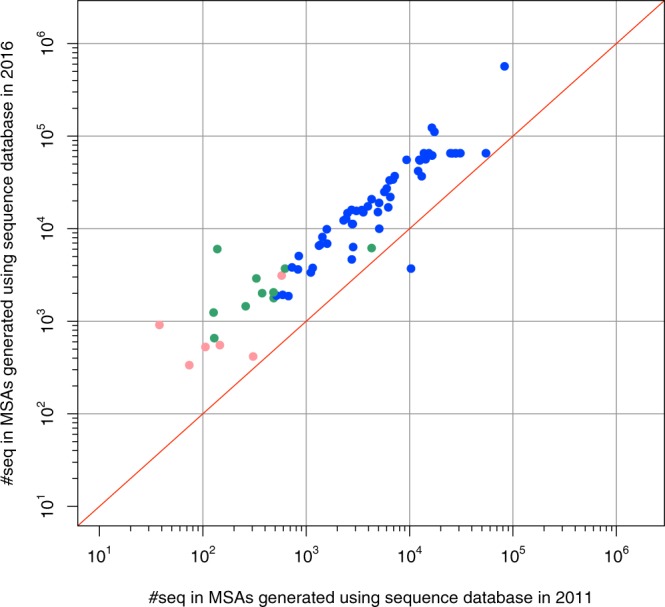
Comparison of the numbers of sequences included in MSAs. The X-axis shows the numbers of sequences in MSAs generated using the sequence database of 2011; the Y-axis shows those using the sequence database of 2016. Monomers in the Duarte dataset were compared. Dots show each monomer. Blue dots represent both 2011 and 2016 MSAs satisfying the diversity requirement of PSICOV. Green dots represent only 2016 MSAs satisfying the requirement. Pink dots represent neither 2011 nor 2016 MSAs satisfying the criterion.

The rapid accumulation of publicly available sequence data has supported large-scale analyses. Particularly, the prediction of contact sites via strong CSs is currently a standard step in the field of *de novo* protein structure prediction. One shortcoming of contact prediction using CSs is that it requires deep alignment^27,32^. As one example, Protein Sparse InverseCOVariance (PSICOV)^24^ filters out non-promising MSAs with the diversity criterion: MSA must contain at least as many non-redundant sequence clusters as the query length. It is invariably an important factor for current contact prediction methods. However, this difficulty has been diminishing during this genome-rich era. To illustrate this phenomenon, we compared the numbers of sequences in the MSAs, which can pass the PSICOV criterion, generated using databases of 2011 and 2016. We collected homologous sequences and generated MSAs using HMM methods: HHblits^34^ and jackhmmer^35^, as described in the *Methods* section. As an example, 92% of monomers in the Duarte dataset^17^ passed the PSICOV criterion when using sequence databases of 2016, whereas 78% of the monomers passed the criterion when using sequence databases of 2011 (Fig. 1). The sequence database growth rate is quite high. Therefore, the quality and feasibility of contact prediction methods using CSs are apparently sufficient for interface classification purposes. Now is the right time to demonstrate the applicability of CS in this field.

We also verified the viability of our approach based on CSs for three existing datasets under the PSICOV criterion: the Duarte, Bahadur, and Zhu datasets. Our results confirmed that most (>84% (or more)) cases in the datasets are analyzable using CS under the PSICOV diversity criterion (Table 1). Preparation of MSAs for hetero-oligomers is still more difficult than that for monomeric or homo-oligomeric structures for the following reasons: (1) Orthologs cannot be discriminated explicitly from paralogs, especially in eukaryotic sequences. (2) Even prokaryotic sequences, for which operon information is applicable, present situations in which only insufficient ortholog sequences are available. (3) Two genes are occasionally related to the same sequences, suggesting recent duplication. The Zhu dataset includes numerous hetero-oligomeric structures. Therefore, its rate of applicability is slightly lower than those of the other two datasets. Nevertheless, generally speaking, numerous interfaces are analyzable using current sequence databases.

**Table 1:** Number of interfaces in each dataset and PSICOV criterion passing rates.

Data source	Biological	Crystallographic	Applicable portion
Duarte *et al*.	72 (83)	76 (82)	89.7%
Bahadur *et al*.	105 (121)	170 (185)	89.9%
Zhu *et al*.	59 (74)	93 (106)	84.4%

Numbers in cells represent the numbers of instances that passed the PSICOV criterion. Numbers in parentheses in cells are total numbers of respective classes in the datasets.

### Contact prediction methods using CSs

Contact pairs in protein–protein interaction interfaces are quantified by the CS computed from these large MSAs. Two widely used approaches exist for distinguishing direct coupling sites from indirect coupling sites: Direct Coupling Analysis (DCA) based on the Potts model through maximization of pseudo-likelihood function^26^ and the inverse of the covariance matrix such as an implementation of PSICOV^24^. We used CCMpred as an implementation of the Potts model in this study^36^. For the inverse of the covariance matrix approach, we used PSICOV^24^.

DCA estimating direct couplings via maximization of pseudolikelihood is currently the most powerful method. Therefore, we expected greater potential of this approach than that of inverse of covariance matrix methods. It remains unknown whether an appropriate threshold exists to discern biological and crystal contacts. Therefore, we attempted several thresholds for the two methods. Results demonstrated that, in terms of the *F*-score, which indicates the segregation of two datasets, PSICOV scores distinguish contact pairs better than CCMpred scores do. In the case of PSICOV, biological interfaces from crystal ones were segregated best at 0.4 and 0.6 (Supplementary Fig. S1). Similarly, discrimination by the CCMPred scores showed the best performance at the threshold, 0.3 (Supplementary Fig. S1). The scores are the *L*_*2,1*_ norm and the *L*_*2,2*_ (Frobenius) norm of the 21 × 21 submatrix, respectively, in PSICOV and CCMpred. In both methods, norms are corrected by the Average Product Correction (APC) to remove a false background signal generated by random noise and/or shared ancestry^37^. Time complexity of PSICOV is independent of the number of sequences, but it depends on the MSA length. Thereby it is sufficiently fast for most samples in this study. Because of its good computational time and performance, we applied PSICOV for this study.

### CSs in biological contact pairs are weak but useful

**Figure 2:**
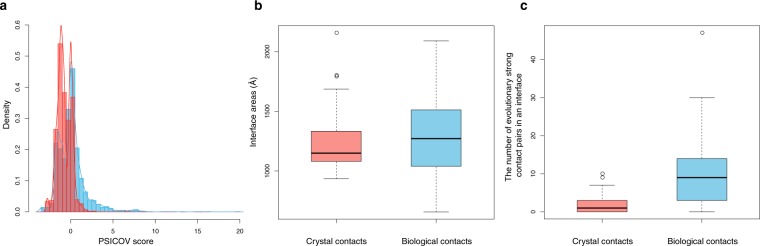
Differences of CSs between biological (blue) and crystal (red) contacts. (**a**) Distributions of PSICOV scores. Filtered pair (indirect coupling sites) are omitted. (**b**) Whisker plot of interface areas for the crystal and the biological contacts in the Duarte dataset. (**c**) Whisker plot of the number of interface contact pairs with PSICOV scores higher than the threshold: 0.4.

Although a large overlap exists between the PSICOV score distributions of biological and crystal contacts (Fig. 2a), the numbers of pairs in the two types of interface seem to have discriminative power if one sets a certain level of CS score threshold (Supplementary Fig. S1). It is noteworthy that the Duarte dataset is a carefully adjusted set by area size between biological and crystallographic interfaces. Therefore, both the interface area and contact pairs in an interface are not significantly different between the two classes (Fig. 2b and Supplementary Fig. S2a). Results show that the number of pairs having a higher CS score than a certain threshold in biological contacts is not related to the area size difference.

Moreover, the CS scores of interchain contact are apparently lower than those of intrachain contacts. We confirmed this trend using a relative ranking of scores normalized by alignment length (Supplementary Fig. S2b). In the top *L/*1 range, where *L* represents the length of the amino acid sequence, only 4.6% of interchain contact pairs on the biological contacts are ranked; 57% of those contact pairs have no PSICOV score. In the top *L* ranks, intrachain contact pairs are monopolized in terms of CS scores.

Nevertheless, quantification of the contact pairs using CS is apparently promising for the classification of contact pairs into covarying and non-covarying pairs. In fact, the numbers of covarying contact pairs are significantly different between biological and crystal contacts (Fig. 2c, *p*-value = 4.56e-12, Mann–Whitney U test). The CS score of interchain biological contact pairs is subtle, but it has discriminative power.

### Effects of sequences included in MSA

The CSs seem to have some capability of discriminating biological contacts from crystallographic interfaces. One simple relevant question is whether estimation of the CS can be improved further through enlargement of MSAs. A tendency by which a larger MSA simply improves coupling methods is well observed in prediction of the intrachain contacts. It would be interesting to elucidate how the number of sequences and divergence among detected sequences affect CS distributions for interchain contacts. To explore this point, we produced a dataset including more sequences using a higher E-value threshold (e-4 > e-20) in the HHblits. In addition, two other datasets were prepared using more stringent thresholds: e-40 and e-60. The number of applicable MSAs for contact prediction is reduced because of reduction of the number of sequences in MSAs if a more stringent threshold is applied. Therefore, all datasets include queries that have a sufficient number of sequences, even at e-60. Other queries were removed from this analysis.

As might be expected, the number of sequences fundamentally increases if a higher E-value threshold applies (Supplementary Fig. S3a). However, inclusion of more sequences does not necessarily engender better discrimination using CSs. Clear improvement was found between the datasets using e-20 and e-40, but the dataset using e-4 showed less discriminative power for interchain contacts (Supplementary Fig. S3b).

Although the number of sequences in MSAs is apparently an important factor, it alone cannot explain this result. As Duarte *et al*. discussed, interchain contacts are conserved differently from intrachain contacts^17^. A threshold for controlling sequence similarity is an important factor when collecting homologous sequences for interface classification. Consequently, in the case of interchain contact prediction, it is better to collect homologous sequences more if and only if an appropriate threshold for interchain contacts is maintained (e.g. e-20 in this case).

### Classification using the CS

**Figure 3:**
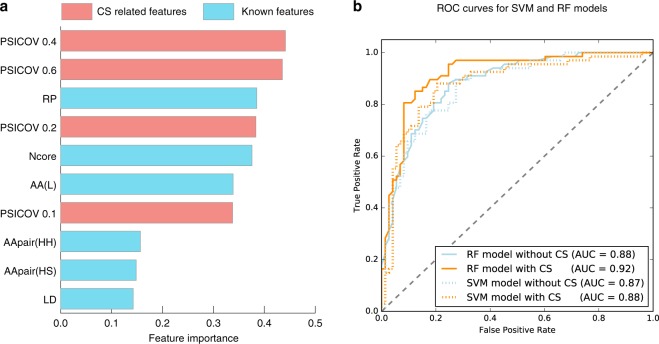
Feature and performance comparisons. (**a**) Feature importance quantified and ranked by F-score. (**b**) Receiver operation characteristic (ROC) curves of classifiers. Blue lines show the classifier performance using selected known features but the CS feature. Orange lines show that of the classifiers using both selected features and the CS feature. Solid lines show performances of RF models, whereas dashed lines show those of SVM models.

Although high sequence similarity is apparently an important factor for inter-protein contact prediction, the number of sequences is a major factor influencing CS of protein interaction pairs. By virtue of recent sequence data augmentation, this difficulty is expected to be improved gradually. However, despite weak signals of inter-protein CS compared to those of CS of intra-protein pairs, we regarded the CS of inter-protein pairs as a promising feature to resolve contact classification difficulties. We simply defined the number of contact pairs that have PSICOV scores higher than a given threshold as a feature. We used four thresholds so that a classifier automatically handles this problem more flexibly. We trained SVM and RF models using known features (see *Methods* for details) or both those and the CS features. Because few training datasets are available, feature selection was conducted to reduce the parameters of classifiers and to increase the generalization capability. To discuss the applicability of the CS features, feature selection was conducted within the known features. Consequently, 32 features were selected from the known features (Supplementary Table S1). The 10 most important features are presented in Fig. 3a. The CS features are ranked highest as the most important features.

The RF model using both the CS features and the 32 known features showed better performance than the model without the CS features (Fig. 3b). Actually, SVM shows similar performance by cross-validation tests. However, performance results obtained for the respective SVM models were slightly worse than those of the RF model using both. Performance improvement by CS features was observed for each of the SVM models and RF models (Fig. 3b), suggesting that adding CS features can enhance the model capabilities.

Next, we tested the RF model performance using both the CS and the known features for the three datasets. Results are presented in Table 2. The RF model showed 82% more sensitivity and 88% more specificity. The model showed 85% or greater accuracy for the three datasets. For these datasets, the MCC of the model was 0.7 or more. The Bahadur and Zhu dataset results were better than those for the Duarte dataset, which was introduced to overcome area dependency in this field^17^. Therefore, the other two datasets include clearer differences in the area size distributions between biological and crystallographic interfaces. Although our model does not explicitly incorporate the interface area, smaller areas are unlikely to include numerous contact pairs, leading to a small number of CS features. Bahadur *et al*. intentionally selected large crystallographic interfaces at that time. Therefore, this set is apparently more difficult than the Zhu dataset. Different performances for three datasets are apparently a result by which the classifier correctly detected differences in the properties. In addition, performance improvements attributable to CS features were observed in all three datasets (Table 2 and Supplementary Table S2).

**Table 2:** Classification performance of the RF model using CS features.

RF model using CS features				
	Sensitivity	Specificity	Accuracy	MCC
Duarte (5-fold c.v.)	82%	89%	85%	0.7
Bahadur	83%	95%	90%	0.79
Zhu	88%	98%	94%	0.87

### Comparison with other classifiers

We compared the results of our RF classifier with state-of-the-art classifiers and widely used ones: PRODIGY-CRYSTAL^23^, EPPIC^17^, PISA^8^, and NOXclass^18^. The same three datasets were used for comparisons: Duarte, Bahadur, and Zhu. The Duarte dataset was used for training in EPPIC and for five-fold cross-validation in our method. The results are presented in Table 3. Similar trends related to better results for the Bahadur or Zhu datasets than those for the Duarte dataset were observed for all methods reflecting the difficulty of the respective datasets. Generally speaking, our RF model shows better overall performance for all three datasets, except for its measurement of sensitivity, compared with the existing three classifiers. NOXclass shows particularly good performance for the Zhu dataset, but it is noteworthy that NOXclass was trained on the Zhu dataset. Those results demonstrate that our proposed method is more accurate than existing methods, although the model sensitivity yields mixed results. It is noteworthy that the model sensitivity is higher than those of EPPIC and PISA for the Zhu dataset, and higher than that of NOXclass for the Duarte dataset. We used smaller datasets than those used in studies described by earlier reports^17^ because of the lack of a large MSA for some samples and because of the removal of sequence redundancies. Nevertheless, the benchmark results are comparable. For the reasons described above, extraction of the subset from the original dataset was more or less arbitrary. Unfortunately, the lack of sufficient homologous sequences in some families is unavoidable at present. Nevertheless, it seems that the continuing expansion of sequence databases will alleviate this problem eventually.

**Table 3:** Classification performance of other representative classifiers.

PRODIGY-CRYSTAL				
	Sensitivity	Specificity	Accuracy	MCC
Duarte	94%	55%	74%	0.53
Bahadur	93%	85%	88%	0.77
Zhu	93%	91%	92%	0.83
**EPPIC (UniRef2016_07)**				
	Sensitivity	Specificity	Accuracy	MCC
Duarte (training)	90%	71%	80%	0.59
Bahadur	89%	86%	87%	0.74
Zhu	86%	97%	93%	0.84
**PISA**				
	Sensitivity	Specificity	Accuracy	MCC
Duarte	91%	59%	74%	0.52
Bahadur	89%	73%	80%	0.62
Zhu	84%	90%	88%	0.74
**NOXclass**				
	Sensitivity	Specificity	Accuracy	MCC
Duarte	69%	62%	65%	0.3
Bahadur	86%	71%	78%	0.57
Zhu (training)	97%	98%	97%	0.94

### Evaluation using a large-scale dataset

We further assessed our RF classifier using another independent dataset having about 6000 interfaces prepared for a large-scale comparison^38^. 84% of the interfaces in the dataset satisfied the PSICOV criterion. We used this subset for further analysis (Supplementary Table S3). The number of target entries is tremendous. For that reason, we specifically examined the most competitive predictors for the comparison: PRODIGY-CRYSTAL, and EPPIC. Regarding the evaluation of PRODIGY-CRYSTAL, 10-fold cross-validation was conducted because this classifier was trained on the same dataset^22^.

In addition, the effect of data augmentation on our RF model was assessed by merging the Duarte, Bahadur, and Zhu datasets. We also assessed our RF model and features on the large-scale dataset using cross-validation. The results are presented in Table 4. Our RF model shows an overall better performance in the large-scale dataset either. Sizes of training datasets are consistent with improvements (Table 4). Although a small degree of overlap exists between the merged and the large-scale test detests, the performance difference was negligible after removal of the overlapped entries (Table 4).

**Table 4:** Classification performance of our RF models, PRODIGY-CRYSTAL, and EPPIC based on Uniref100 (2016_07).

Performance on the large-scale dataset				
	Sensitivity	Specificity	Accuracy	MCC
RF model^*1^	90%	93%	91%	0.82
RF model^*2^	92% (92%)	94% (94%)	93% (93%)	0.86 (0.86)
RF model^*3^	95%	96%	95%	0.91
PRODIGY-CRYSTAL^*4^	91%	94%	92%	0.85
EPPIC	90%	88%	89%	0.78

*1: The model was trained on the Duarte dataset, which is the same model described in Table 2 exploiting CS features. *2: The model was trained on the merged datasets (the Duarte, Bahadur, and Zhu datasets were merged). Numbers in parentheses represent performance on the dataset having no overlapped entries, where 35 overlapped entries between the merged and the large datasets were removed. *3: The model was trained and evaluated using the large-scale dataset by applying 10-fold cross-validation. *4: Because the classifier was trained on the same dataset, 10-fold cross-validation was conducted. For our RF model and PRODIGY-CRYSTAL, the same partitioning was applied for each fold.

## Discussion

After verifying the applicability of contact prediction methods using CSs in crystal interface classification problems, we proposed a novel method that is slightly more accurate than existing methods. It discriminates biological interfaces from non-biological ones in protein crystal structures by exploiting subtle CSs derived from MSA using a sophisticated contact prediction method. To elucidate the benefits of our method, we applied it to cases examined in earlier research. One interesting example is pyrrolidone-carboxylate peptidase^7^ (PDB id: 1IU8). Proteins of this family are fundamentally crystallized in a tetramer form, but an asymmetric form of 1IU8 is a homodimer. The functional form of this family is controversial^39^. Our method, PRODIGY-CRYSTAL, and PISA (and PQS also) predict this as a tetrameric protein (Supplementary Fig. S4). Furthermore, PiQSi^40^, the human-curated database for oligomeric state, annotates this as a tetramer. It is difficult to ascertain the true oligomeric state conclusively. However, our method was capable of classifying three interfaces (within asymmetric unit and between asymmetric unit and crystal symmetry mates) as biologically relevant ones, although two other classifiers (EPPIC and NOXxlass) show this protein as a monomer.

Our analysis revealed that contact pairs in biological interfaces often display weak CS in comparison to intrachain contact pairs. Recently, Talavera *et al*. elucidated why a large MSA is necessary to predict coupling sites in the context of a “coevolutionary paradox”. According to their observation, successful intrachain prediction methods for contacts detect highly conserved sites with fewer substitutions, which tend to constitute the core of the structure^41^. In contrast to the detectable highly conserved sites, interacting pairs are located on the surface in the interface classification problem. If covariance methods tend to detect slightly variable but conserved sites, then weak signals of interchain contacts are explainable by different levels of conservation. This inference is consistent with the notion that CS estimation for interchain contacts requires a greater number of similar sequences (Supplemental Fig. 3b). Our method appears to be the first and most comprehensive that applies such subtle CSs explicitly to interface classification, although successful examples in earlier research probably used strong CSs^30,32,33^.

As we discussed above, relationship between sequence similarity and contact prediction capability seems to vary upon the local environment of contacts. During the review period, another group tackled the crystal interface classification problem by a different formulation of the CS^42^. One of major differences is a way to collect homologous sequences. They applied PSI-Blast at a relatively lenient threshold (e-value < 0.001). As shown in Supplementary Fig. S3b, the effect of sequence divergence is not ignorable for intermolecule contacts. Under our framework, we assessed the effect of sequence search algorithm and parameters applied in their approach; consequently, MSA constructed by their parameters showed less discriminative power (Supplementary Fig. S5, Table S4). Because the number of sequences is a vital factor for contact prediction, future works should consider this importance.

If local environment is important for contact prediction, are there other factors to explain CS difference more in details? Additionally, we compared physico-chemical properties of contact pairs to ascertain whether they are correlated with CSs (Supplementary Fig. S6). Hydrophobic interaction pairs seem to have higher CSs. Although the difference is not clear (statistically significant at α = 0.05), hydrophobic interaction pairs have significantly higher scores than pairs without annotations (Supplementary Table S5). The CS scores of contact pairs that have hydrogen bonds do not show a statistically significant difference from other groups. It is noteworthy that the pairs between the core residues tend to have slightly higher CSs than the others. However, the difference was not significant. This tendency was not observed for crystal contacts (Supplementary Fig. S6 and Table S5). The conservation of interactive surfaces has been discussed. Interacting residues are generally conserved^15,17^. In intrachains, a contact pair between conserved residues located in a hydrophobic environment shows strong CS^41^. Subtle CSs in interchain contacts do not contradict such a discussion in intrachain contacts.

**Figure 4:**
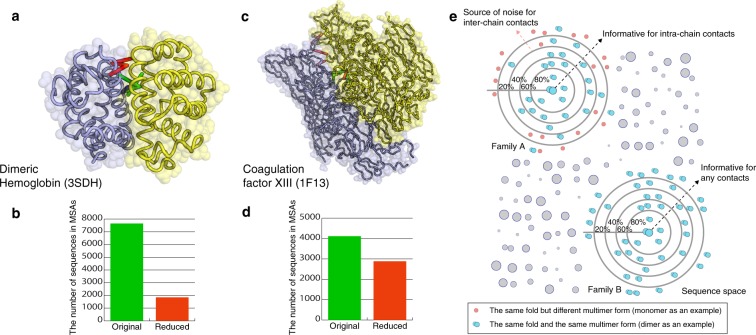
A practical example of our method and enhanced CSs in contact pairs of homodimers in reduced MSAs. (**a**) Enhanced CSs in contact pairs of homodimeric hemoglobin. Red bars show contact pairs with CS higher than 0.6 when using the reduced alignment. Blue bars show those when using the original alignment. Green bars show contact pairs having CS higher than 0.6 in both alignments. (**b**) Number of sequences included in MSAs of 3SDH with the default (green) and the conservative (red) thresholds. (**c**) Enhanced CSs in contact pairs of human coagulation factor XIII. Color scheme is the same as 3SDH. (**d**) Number of sequences included in MSAs of 1F13. Color scheme is the same as 3SDH. (**e**) Schematic diagram of differences in contact prediction of interchain and intrachain interactions. Required sequences for the estimation of CS depend on the degree of conservation in target contacts. In general, CS estimation of interchain contacts requires more similar sequences because of their lower degree of conservation of oligomeric states compared to folds. Although intrachain contacts are more conserved (i.e., even quite diverged sequences are still informative for intrachain contact estimation), interchain contacts are less conserved because oligomeric states can vary more than folds. This is at least true for our manually confirmed samples. In such cases, greatly diverged sequences, which accommodate different (oligomeric) states from that of the target, can be a source of noise in the estimation. The sequence threshold for these samples is apparently appropriate at 20–40%. Family A in the figure illustrates a protein family where diverged sequences can be noise. In contrast, even diverged sequences are still informative for interchain contacts if oligomeric state is highly conserved. Family B is an example for this case.

The degree of conservation is an important factor for interface classification using CS estimation: fold is conserved more than in oligomeric states^43^. We can discuss this matter carefully by presenting the following two examples from the Bahadur dataset. One example is the globin family. *Scapharca inaequivalvis* has two forms of hemoglobin: homodimeric HbI and hetero-tetrameric HbII^44^. The Bahadur dataset includes HbI (PDB id: 3SDH). Its interface is predicted as a crystallographic one with a score close to the threshold by the default setting of our classifier because of a few strong signals. PiQSi annotated that 3SDH is a dimer and that the same SCOP family (= globins) of 3SDH includes monomeric proteins, myoglobin. Consequently, large alignment for 3SDH includes similar structures but different oligomeric state sequences, which can be a source of noise in this case. Even with a conservative threshold, e-20, the MSA included myoglobin sequences. We removed most distant sequences, in terms of sequence identity, from the 3SDH MSA while maintaining the PSICOV diversity criterion to polish the MSA. Therefore, some interacting pairs in the interface gained higher CSs than the MSA with more sequences (Fig. 4a,b). The interface was predicted as a biologically relevant one because of the higher values in the contact features. A similar example is Coagulation factor XIII (PDB id: 1F13). According to the annotation of PiQSi, the family of 1F13 includes monomeric proteins such as a glutamine transferase. Although the interface of 1F13 is predicted to be a biological one by the default setting of our classifier, the polished alignment showed higher CSs (Fig. 4c,d), which led to a higher prediction score. These phenomena are consistent with the conservative threshold used to collect homologous sequences in EPPIC^17^.

As one of main challenges in this field, the number of interfaces in training/testing datasets should be argued. In fact, the number of interfaces passed the PSICOV criterion in the Duarte dataset is 140 interfaces in total, and those in the Bahadur and the Zhu datasets are 241 and 146 interfaces, respectively. Amongst the three datasets (the Duarte, the Bahadur, and the Zhu datasets), there are 22 overlapped biological interfaces between the Bahadur and the Zhu datasets. Therefore, the number of actual testable interfaces can be even smaller. The Duarte dataset has no overlap with either the Bahadur or Zhu datasets; therefore, bias should be small. However, it is ideal to assess a method in a larger scale for a better statistics. Because the large-scale dataset was introduced by Baskaran *et al*.^38^, we also tested our approach on this large-scale dataset. Our RF model using CS features also shows an overall better performance against the large-scale dataset (Table 4). It is also noteworthy that our model showed even better performance against the large-scale dataset if we include Bahadur and Zhu datasets for training (Table 4). Although there are 35 overlapped entries between the merged and the large-scale dataset, removal of those entries did not affect the results significantly (Table 4). Inclusion of the two datasets augmented the training set for interface classification (140 to 527 entries); as a result, it seems that the predictor learned a better model. In addition to training data augmentation by merging, we also evaluated and confirmed the applicability of our model and features on this dataset by the cross-validation (Table 4). Because each fold of this large-scale dataset gives even larger training data (about 4300 entries). Therefore, it seems that performance improvement was observed by expansion of the training space.

Contact prediction for intrachain contacts often uses an extremely rough threshold to collect homologous sequences because a larger MSA fundamentally engenders higher performance^45^. However, for the interface classification problem, this generalization is true if and only if target contacts are conserved as discussed above (Fig. 4e). For cases with contacts (oligomeric states) that are not conserved even in their families, polishing MSA is useful to reduce noise and to enhance the CSs of the biological interface. Unfortunately, because of the tradeoff between enhancement of the signal and purification of oligomeric states in contact prediction methods, polishing MSA is not simple. The former seems to require more diverse sequences to detect variations. However, the removal of distant homologous sequences with oligomeric states that can be different reduces diversity. Overly strong polishing engenders loss of diversity, in turn leading to weakened signals. In the case of 3SDH, polishing of MSA enhanced the CSs of contact pairs. However, further polishing of the MSA weakened CSs again. Optimal diversity of MSA for the interface classification problem is expected to vary according to the degree of conservation of oligomeric states. Developing methods such as those devised for functional region prediction^46–48^ for selecting appropriate homologous sequences will be important future work to detect the interchain contact pairs correctly.

## Methods

### Datasets

#### Training set

The Duarte dataset^17^ was used for the analyses presented in Figs 1–3, and for training classifiers. Structures for which MSA did not fulfill the diversity criterion of PSICOV were excluded from the dataset. This exclusion left 72 and 76 interfaces, respectively, for biological and crystallographic datasets. Sequence redundancy was removed for training at the 30% identity level. Finally, 67 and 73 non-redundant interfaces remained, respectively, in biological and crystallographic datasets. These 140 interfaces were used for five-fold cross-validation and comparisons with other standard methods.

#### Testing sets

For independent tests, Bahadur^9,49^ and Zhu^18^ datasets were also applied. For crystal contacts, the largest crystal interfaces were extracted from monomers in Bahadur and dimers in Zhu datasets. ‘Obligate’ interaction and ‘crystal’ sets in the Zhu dataset were used for this study. As biological interfaces, the largest were selected from each entry in the Bahadur and Zhu dimer datasets. The PSICOV diversity filter was also applied for MSAs in each dataset. We obtained 105 and 170 interfaces, respectively, as biological and crystallographic in the Bahadur dataset. We extracted 59 and 93 interfaces, respectively, for biological and crystallographic interfaces from the Zhu dataset. Sequence redundancy was removed at the 30% identity level within each dataset and against the training datasets. Consequently, 103 biological and 138 crystallographic interfaces remained. Similarly, 58 biological and 88 crystallographic interfaces remained in the Zhu dataset. The same subsets applied for testing and comparisons. It is noteworthy that non-obligate complexes in the Zhu dataset were not included in this study because, in the clear majority cases, the multiple sequence alignments were too sparse to be analyzed. In addition to these datasets, another large-scale dataset^38^ was applied. The same PSICOV criterion was considered for this dataset either. As a result, 2299 and 2525 interfaces were retained for biological and crystallographic interfaces. We also assessed the model, which is trained on the Duarte, the Bahadur, and the Zhu datasets. Because there are 35 overlapped structures between the large-scale dataset and the other datasets, the large-scale dataset having no overlapped entries was also evaluated.

### MSA construction

Multiple sequence alignments for monomeric and homo-oligomeric structures were generated using, at most, eight iterations of HHblits ver. 2.0.15^34^ against uniprot20_2016_02. If an MSA from HHblits did not pass the PSICOV sequence diversity criterion, then an MSA generated using, at most, eight iterations of jackhmmer in HMMER ver. 3.1^35^ against UniRef100 (2016_07) was also applied. For hetero-oligomeric structures, if two cleaved chains are derived from the same gene, then the same procedure was applied. Otherwise, orthologous pairs of hetero-oligomers were built by the GREMLIN server^32^ using HHblits. For comparison of the number of sequences in MSAs generated using older databases in 2011, nr20_12Aug11 and UniRef100 (2012_01) were used respectively for HHblits and jackhammer. Only one archived version of UniRef100 (2011_01) in 2011 was apparently too old to be compared. Therefore, the oldest version in 2012 was applied instead.

### Contact prediction based on CSs

To quantitate interaction pairs in interfaces, we applied two widely used unsupervised contact prediction methods: PSICOV^24^ and CCMpred^36^. A default sequence clustering threshold was applied to each method. If default sequence clustering thresholds failed to pass the diversity criterion of PSICOV for MSAs from both HHblits and jackhmmer, then the threshold was increased by 1% increments up to 80% or until the criterion was met. Although phylogenetic bias correction similar to APC^37^ was computed in PSICOV, this correction was modified similarly to an earlier work^32^ to accept hetero-oligomeric MSAs.

### Surface and interface definition

The solvent accessible surface area (SASA) of a query molecule was calculated using the Shrake–Rupley algorithm^50^. The rolling ball radius and the number of probe spheres were, respectively, set as 1.4 (Å) and 3000. Neighboring asymmetric units were reconstructed to find crystal symmetry mates for the query molecule. The buried surface area (BSA) of each residue was computed by subtracting the SASA of the residue with an interacting partner from SASA of residue without other subunits in the complex. An interface is defined if there is at least one non-hydrogen atom-pair with distance of less than 5.5 Å between the query and the interacting partner. Both such atoms shall have at least 0.1 Å^2^ BSA. The residue contact pair on an interface is defined as a pair fulfilling the following conditions: (1) both residues have all main-chain atoms (no ambiguous residue was allowed); and (2) both residues are surface residues. Here, we used the definition of surface residue that has relative SASA higher than 25%^51^. It is noteworthy that our method is robust to the difference of relative SASA thresholds. However, 25% shows the best performance (Supplementary Table S6).

Among interface residues, fully buried residues are defined as core residues for an interface. Our definition of the core residue of an interface was suggested in an earlier report^16^: a surface residue with BSA/SASA > 0.95.

### Feature computation

#### Amino acid composition on an interface (AA)

Total BSA for 20 amino acid residues. The BSA value for each amino acid is divided by the total BSA of the interface.

#### Amino acid composition of the core residues (AAc)

The computation is the same as that above. However, only the core residue of an interface is considered. BSA values for the respective amino acids are divided by the total BSA of core residues.

#### Amino acid pair frequency (AApair)

The pair frequency of amino acids was computed from contact pairs of an interface. Using 400 parameters in naive combination to reduce dimensions, we grouped 20 amino acids into six groups: small (A, C, G, P, S, T), negatively charged (D, E), positively charged (H, K, R), aromatic (F, W, Y), hydrophobic (I, L, M, V), and other (N, Q) residues.

#### Local density index (LD) and Residue Propensity score (RP)

Averaged atomic density for an interface and sum of the propensity for amino acid of all types, which respectively appeared on an interface. Details were presented in an earlier report^9^.

#### Gap Volume Index (GVI)

Gap volume was computed using SurfNet implemented in UCSF Chimera^52^. Those numbers are divided further by the total BSA of an interface^18^.

#### Ncore

The number of core residues defined in an earlier study^16^.

#### CS-score

Contact pairs with CS higher than a given threshold within an interface are simply counted (*e.g*. PSICOV 0.2 denotes that the threshold is 0.2). Although the definition of the contact pair is described above, we filtered contact pairs fulfilling a few more conditions to avoid artifacts: (1) at least four residues exist between two residues in a primary structure (positional difference must be greater than 5); and (2) neither residue is internally proximal. Consequently, the distance between Cβ atoms of two residues is greater than 8 Å. Because of these conditions, we infer that such contact pairs show higher CSs because of their interchain proximity.

All features presented above are computed mainly using our code based on BioJava 4.2.3^53^. From the full set of features, 32 features were selected (Supplementary Table S1).

### Intermolecular interactions

Post-hoc analysis between CS-score and physicochemical molecular interactions was conducted following detection of the physicochemical interactions using IChem toolkit^54^. The contact pairs (described in the Feature definition) were classified using IChem toolkit as having hydrogen bond, ionic, aromatic, pi/cation, or hydrophobic interaction. Pairs having multiple interaction annotations were removed from analysis because which factor has affected the evolutionary history is not clear in a case of multiple annotations. Hydrophobic interactions were further grouped into three categories as rim–rim (R/R), core–rim (C/R), and core–core (C/C), respectively, where both pairs were classified to a rim, either residue was classified as the core, and both pairs were classified as the core. Aromatic, pi/cation, and ionic interactions were also considered, but they were excluded from the analysis because of the very few observations in the dataset: fewer than 10 interactions.

### Machine learning algorithms and feature importance analysis

Because of the small training dataset, we applied classifiers that depend on few parameters: SVM^55^ and RF^56^ implemented respectively in LIBSVM^57^ and scikit-learn^58^. The Radial Basis Function (RBF) kernel was applied to SVM. Feature selection was conducted by F-score ranking and SVMs using the RBF kernel^59^. The selected features were also used in the Random Forest (RF) model. ROC curve analysis was performed using scikit-learn and was visualized using matplotlib^60^.

### Benchmarking

We compared our novel classifier with state-of-the-art and widely used classifiers: PRODIGY-CRYSTAL, EPPIC, PISA, and NOXclass. To assess PRODIGY-CRYSTAL, interfaces were classified by the local version^22^ (the source code is available at http://github.com/haddocking/interface-classifier). For the large-scale dataset, we used precomputed features and the model provided with the source code. The feature matrix was modified to fit the model; 10-fold cross-validation was conducted using scikit-learn. Prediction of EPPIC was conducted using the local version with homologous sequences searched in UniRef100 (2016_07). PISA prediction was benchmarked by parsing XML files. The criterion used for PISA prediction is the same as that used in an earlier study^17^. Results of NOXclass were obtained from the web server (http://noxclass.bioinf.mpi-inf.mpg.de/) using its default setting (multi-stage SVM classification model with three features, i.e., interface area, interface area ratio, and area-based amino acid composition). Interfaces that were predicted as obligate interaction interfaces were treated as biological interfaces. Otherwise, interfaces were treated as crystallographic interfaces.

### Software

The classifier is written mainly in Java and is licensed under the GPL. The source code is available at github (https://github.com/yfukasawa/piaco). Outputs of external tools are automatically integrated as an input to the classifier.

### Classification performance evaluation

The prediction performance was evaluated using four measures: sensitivity, specificity, accuracy, and the Matthews Correlation Coefficient (MCC). Sensitivity (Sn), specificity (Sp), and accuracy (Ac) are defined, respectively, as shown below.

$${\rm{Sn}}=\frac{TP}{TP+FN},$$

$${\rm{S}}{\rm{p}}=\frac{TN}{FP+TN},$$

$${\rm{Ac}}=\frac{TP+TN}{TP+FN+FP+TN}.$$

MCC is a measure of performance for binary classification defined as

$${\rm{MCC}}=\frac{TP\_N-FP\_w:N\,}{\sqrt{(TP+FN)\_TP+FP)\_TN+FP)\_TN+FN}}.$$

In the equations above, “T” and “F” respectively stand for “true” and “false”, whereas “N” and “P” respectively denote “negative” and “positive”. Additionally, we use the area under the curve (AUC) of the ROC if a classifier outputs a continuous score. AUC is presented as 0.0–1.0 (1,0 for perfect classification; a completely random classifier is expected to be 0.5).

## Supplementary information

Supplementary Information
