# Supplementary material for: Accurate Classification of Biological and non-Biological Interfaces in Protein Crystal Structures using Subtle Covariation Signals
Source: Sci Rep. 2019 Aug 30;9:12603. doi: 10.1038/s41598-019-48913-8 (PMC6717244; doi:10.1038/s41598-019-48913-8)
Supplement: Supplementary file 1 — Supplementary Information [file 41598_2019_48913_MOESM1_ESM.pdf]

Accurate Classification of Biological and non-Biological Interfaces in Protein Crystal Structures using Subtle Covariation Signals

Yoshinori Fukasawa[1,*] and Kentaro Tomii[1,2,3,*]

[1] Artificial Intelligence Research Center, National Institute of Advanced Industrial Science and Technology (AIST), 2-4-7 Aomi, Koto-ku, Tokyo 135-0064, Japan

[2] Biotechnology Research Institute for Drug Discovery, National Institute of Advanced Industrial Science and Technology (AIST), 2-4-7 Aomi, Koto-ku, Tokyo 135-0064, Japan

[3] AIST-Tokyo Tech Real World Big-Data Computation Open Innovation Laboratory, Tokyo 152-8550, Japan

*corresponding authors: y-fukasawa@outlook.com, k-tomii@aist.go.jp

Supplementary Figures

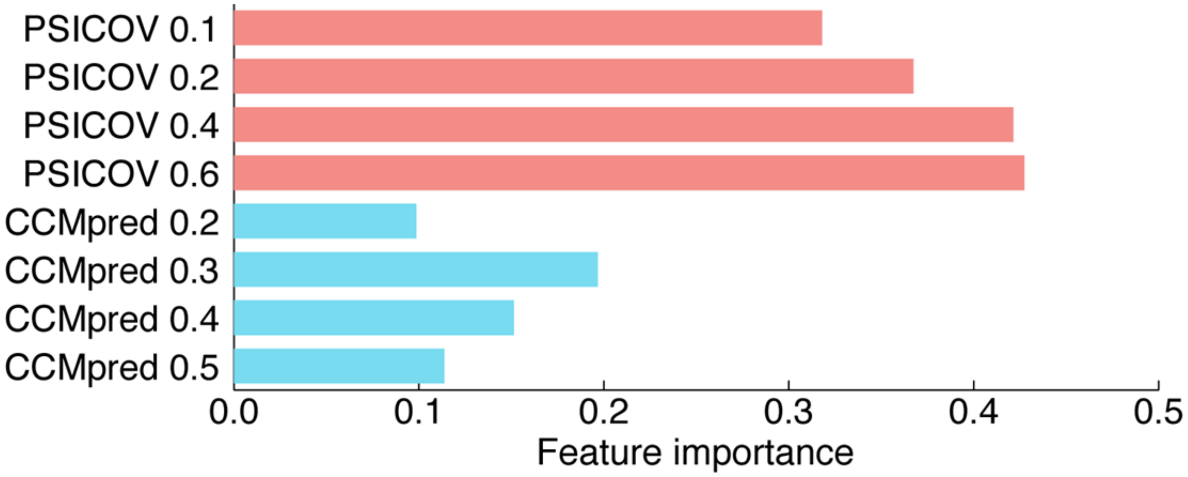

Supplementary Figure S1: Applicability of CSs in two different methods. Feature importance, an overlap of two distributions, is computed by F-score.

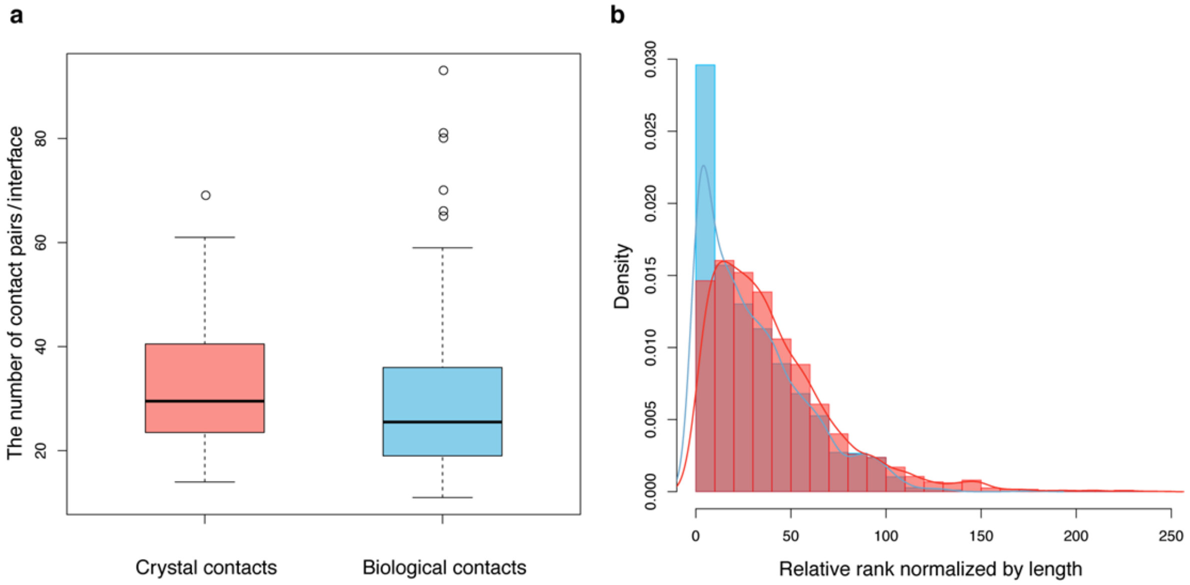

Supplementary Figure S2: Analysis of Duarte dataset using PSICOV scores. (a) Distributions of the number of contact pairs per interface in crystal and biological contacts. (b) Distributions for relative ranks of contact pairs. Pink histogram shows a distribution of relative ranks in crystal contacts, and light blue means that in biological contacts.

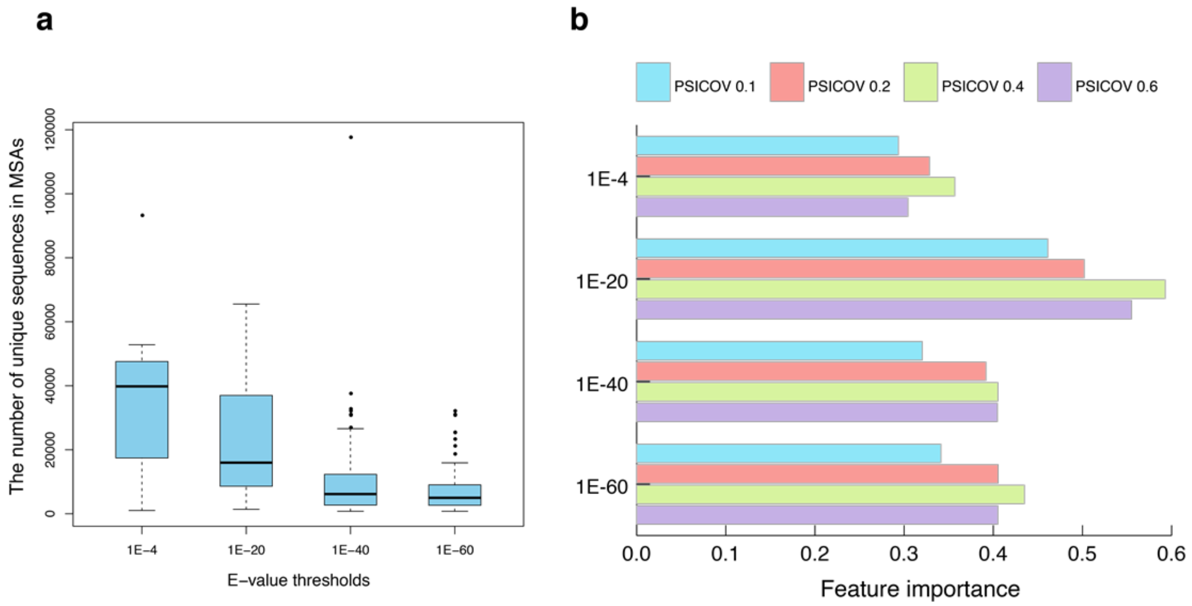

Supplementary Figure S3: Effect of the number of sequences in MSA for PSICOV scores. (a) Comparisons of the number of sequences in MSAs when changing E-value threshold in HHBlits. (b) Effect of sequence gathering thresholds in PSICOV scores. Feature importance is measured by F-score.

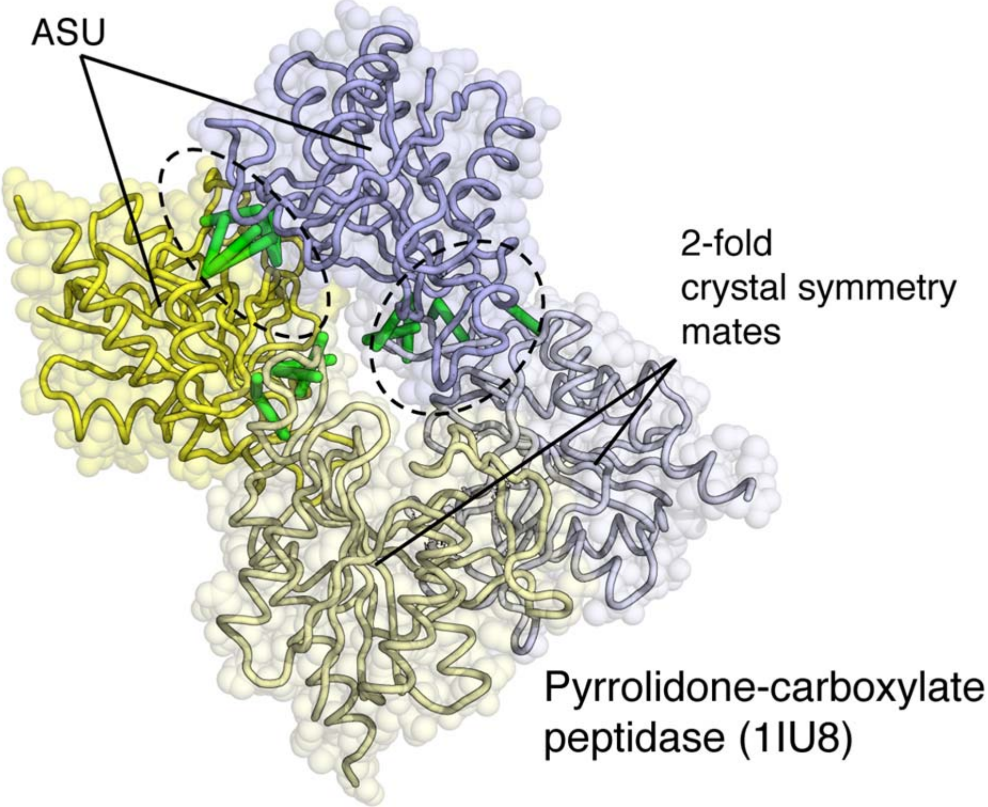

Supplementary Figure S4: A practical example of our method.

Predicted interfaces of 1IU8 as an example. Green bars show contact pairs which have higher CS than 0.6. Dimer generated by a symmetry operation was shown in lighter colors.

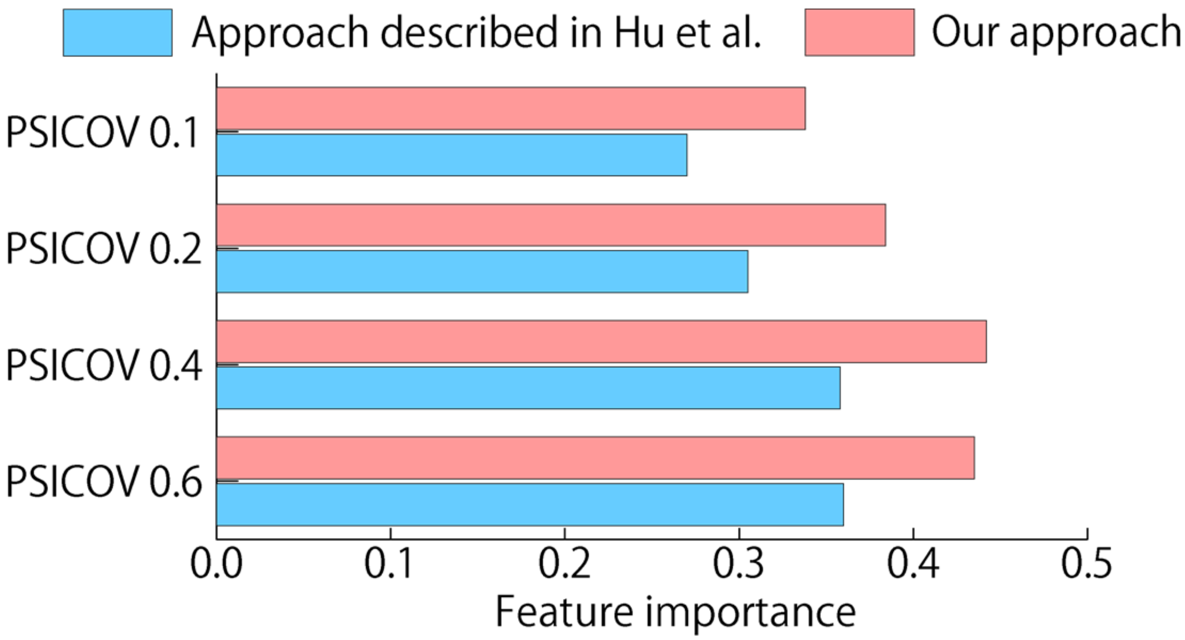

Supplementary Figure S5: Comparisons of the MSA constructed by parameters applied by Hu *et al*[1] and our parameters. Feature importance is measured by F-score.

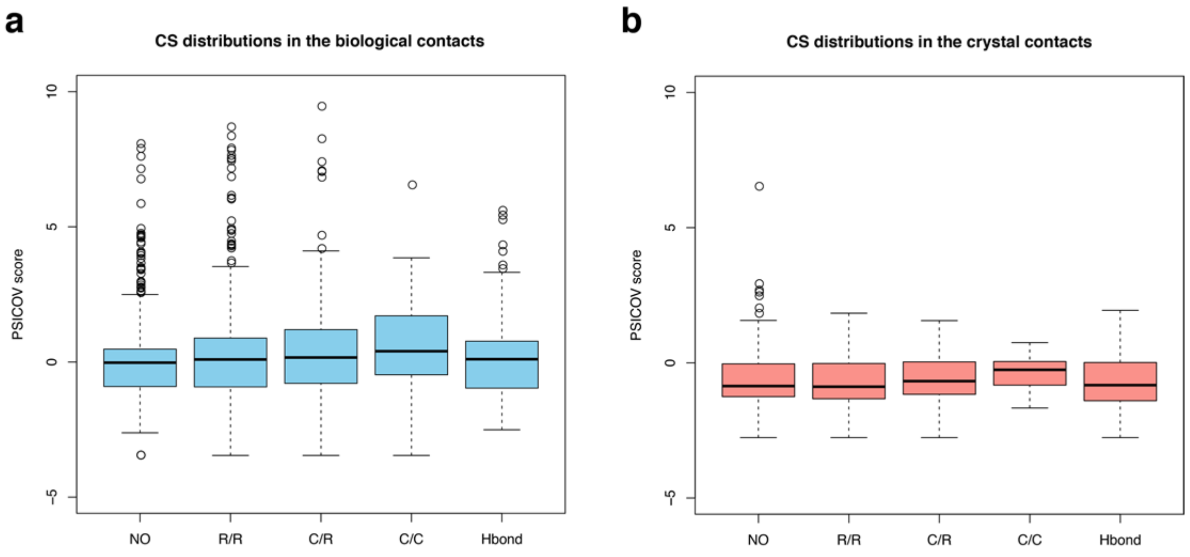

Supplementary Figure S6: CS distribution of pairs in different local environment. (a) CS score distributions in the biological contacts. (b) CS score distributions in the

crystal contacts. NO is the group where pairs have no physic-chemical annotation. R/R, C/R and C/C are groups of contact pairs in hydrophobic environment; moreover, residues of pairs are classified into the core (C) or the rim (R) according to their buriedness. Hbond is the group of pairs having at least one annotated hydrogen bond.

Supplementary Tables

Name	F-score
Known features	
Ncore	0.375897
AA(D)	0.029829
AA(E)	0.138587
AA(F)	0.031724
AA(H)	0.027635
AA(I)	0.085913
AA(K)	0.087255
AA(L)	0.338767
AA(M)	0.060569
AA(N)	0.03877
AA(Q)	0.049614
AA(V)	0.052361
AAc(F)	0.040262
AAc(K)	0.018645
AAc(L)	0.02871
AAc(R)	0.028683
AApair(NN)	0.097839
AApair(KK)	0.067945

AApair(HH)	0.156806
AApair(NO)	0.071782
AApair(PS)	0.031126
AApair(KN)	0.032922
AApair(HK)	0.035547
AApair(OS)	0.050736
AApair(KO)	0.039467
AApair(NS)	0.042034
AApair(HN)	0.020116
AApair(HP)	0.087363
AApair(HS)	0.148463
LD	0.142637
RP	0.385164
GVI	0.049069
CS related features	
PSICOV 0.1	0.337755
PSICOV 0.2	0.383453
PSICOV 0.4	0.441543
PSICOV 0.6	0.435384

Supplementary Table S1: List of the selected features. Letters in parentheses mean one letter amino acid code for AA and AAc feature. Two letters in parentheses of AApair show pairs between amino acids in reduced letters; namely, S (A, C, G, P, S, T), N (D, E), K (H, K, R), P (F, W, Y), H (I, L, M, V), and O (N, Q), respectively. Feature importance is measured by F-score.

RF model using only known features				
	Sensitivity	Specificity	Accuracy	MCC
Duarte (5-fold c.v.)	81%	81%	81%	0.61
Bahadur	79%	91%	86%	0.71
Zhu	74%	93%	86%	0.70

Supplementary Table S2: Classification performance of the RF model using only known features.

Data source	Biological	Crystallographic	Applicable rate
Baskaran et al.	2299 (2832)	2525 (2914)	83.95%

Supplementary Table S3: Number of interfaces in the large-scale dataset and PSICOV criterion passing rate. Numbers in cells represent the numbers of instances that passed the PSICOV criterion. Numbers in parentheses in cells are total numbers of respective classes in the dataset.

**RF models with CS features using PSI-Blast**				
	Sensitivity	Specificity	Accuracy	MCC
Duarte (5-fold c.v.)	82%	84%	83%	0.66

Supplementary Table S4: Classification performance of the RF model with the CS features using PSI-Blast.

	NO	R/R	C/R	C/C	Hbond
NO	NA	0.017	0.002	0.020	1.000
R/R		NA	1.000	0.844	1.000
C/R			NA	1.000	1.000
C/C				NA	0.521
Hb					NA

	NO	R/R	C/R	C/C	Hbond
NO	NA	1.000	1.000	1.000	1.000
R/R		NA	1.000	1.000	1.000
C/R			NA	1.000	1.000
C/C				NA	1.000
Hb					NA

Supplementary Table S5: Comparisons of CS scores in different local environments. Each cell shows *p*-values of Mann-Whitney U tests. The values were corrected by Bonferroni correction.

RF models with different threshold for surface residue				
	Sensitivity	Specificity	Accuracy	MCC
Duarte (5-fold c.v.) rASA 25%	82%	89%	85%	0.7
Duarte (5-fold c.v.) rASA 10%	82%	88%	84%	0.69
Duarte (5-fold c.v.) rASA 5%	79%	86%	83%	0.66

Supplementary Table S6: Classification performance of the RF model using the CS features and different relative SASA thresholds for the surface residue.

1 Hu, J., Liu, H. F., Sun, J., Wang, J. & Liu, R. Integrating co-evolutionary signals and other properties of residue pairs to distinguish biological interfaces from crystal contacts. *Protein Sci* **27**, 1723-1735, doi:10.1002/pro.3448 (2018).
